# Glycemic control is independently associated with rapid progression of coronary atherosclerosis in the absence of a baseline coronary plaque burden: a retrospective case–control study from the PARADIGM registry

**DOI:** 10.1186/s12933-022-01656-9

**Published:** 2022-11-12

**Authors:** Ki-Bum Won, Byoung Kwon Lee, Fay Y. Lin, Martin Hadamitzky, Yong-Jin Kim, Ji Min Sung, Edoardo Conte, Daniele Andreini, Gianluca Pontone, Matthew J. Budoff, Ilan Gottlieb, Eun Ju Chun, Filippo Cademartiri, Erica Maffei, Hugo Marques, Pedro de Araújo Gonçalves, Jonathon A. Leipsic, Sang-Eun Lee, Sanghoon Shin, Jung Hyun Choi, Renu Virmani, Habib Samady, Kavitha Chinnaiyan, Daniel S. Berman, Jagat Narula, Leslee J. Shaw, Jeroen J. Bax, James K. Min, Hyuk-Jae Chang

**Affiliations:** 1 Department of Cardiology, Dongguk University Ilsan Hospital, Dongguk University College of Medicine, Goyang, South Korea; 2 Department of Cardiology, Severance Cardiovascular Hospital, Yonsei University College of Medicine, Yonsei University Health System, Seoul, South Korea; 3 Yonsei-Cedars-Sinai Integrative Cardiovascular Imaging Research Center, Yonsei University College of Medicine, Yonsei University Health System, Seoul, South Korea; 4 Department of Cardiology, Gangnam Severance Hospital, Yonsei University College of Medicine, Seoul, Korea; 5 Department of Radiology, New York-Presbyterian Hospital and Weill Cornell Medicine, New York, NY USA; 6 Department of Radiology and Nuclear Medicine, German Heart Center Munich, Munich, Germany; 7 Department of Cardiology, Seoul National University College of Medicine, Cardiovascular Center, Seoul National University Hospital, Seoul, South Korea; 8 Ospedale Galeazzi-Sant Ambrogio IRCCS, University of Milan, Milan, Italy; 9 Centro Cardiologico Monzino, IRCCS, Milan, Italy; 10 Department of Medicine, Lundquist Institute at Harbor UCLA Medical Center, Torrance, CA USA; 11 Department of Radiology, Casa de Saude São Jose, Rio de Janeiro, Brazil; 12 Seoul National University Bundang Hospital, Sungnam, South Korea; 13 Department of Radiology, Fondazione Monasterio/CNR, Pisa/Massa, Italy; 14 UNICA, Unit of Cardiovascular Imaging, Hospital da Luz, Lisboa, Portugal; 15 Nova Medical School, Lisbon, Portugal; 16 Department of Medicine and Radiology, University of British Columbia, Vancouver, BC Canada; 17 Department of Cardiology, Ewha Womans University Seoul Hospital, Seoul, Seoul Korea; 18 Department of Cardiology, Pusan University Hospital, Busan, South Korea; 19 Department of Pathology, CVPath Institute, Gaithersburg, MD USA; 20 Department of Cardiology, Emory University School of Medicine, Atlanta, GA USA; 21 Department of Cardiology, William Beaumont Hospital, Royal Oak, MI USA; 22 Department of Imaging and Medicine, Cedars Sinai Medical Center, Los Angeles, CA USA; 23 Icahn School of Medicine at Mount Sinai, New York, NY USA; 24 Department of Cardiology, Leiden University Medical Center, Leiden, The Netherlands; 25 Department of Cardiology, Severance Cardiovascular Hospital, Yonsei-Cedars-Sinai Integrative Cardiovascular Imaging Research Center, Yonsei University College of Medicine, Yonsei University Health System, 50-1 Yonsei-ro, Seodaemun-gu, 03722 Seoul, South Korea

**Keywords:** Hemoglobin A1c, Coronary artery disease, Progression, Coronary computed tomography angiography

## Abstract

**Background:**

The baseline coronary plaque burden is the most important factor for rapid plaque progression (RPP) in the coronary artery. However, data on the independent predictors of RPP in the absence of a baseline coronary plaque burden are limited. Thus, this study aimed to investigate the predictors for RPP in patients without coronary plaques on baseline coronary computed tomography angiography (CCTA) images.

**Methods:**

A total of 402 patients (mean age: 57.6 ± 10.0 years, 49.3% men) without coronary plaques at baseline who underwent serial coronary CCTA were identified from the Progression of Atherosclerotic Plaque Determined by Computed Tomographic Angiography Imaging (PARADIGM) registry and included in this retrospective study. RPP was defined as an annual change of ≥ 1.0%/year in the percentage atheroma volume (PAV).

**Results:**

During a median inter-scan period of 3.6 years (interquartile range: 2.7–5.0 years), newly developed coronary plaques and RPP were observed in 35.6% and 4.2% of the patients, respectively. The baseline traditional risk factors, i.e., advanced age (≥ 60 years), male sex, hypertension, diabetes mellitus, hyperlipidemia, obesity, and current smoking status, were not significantly associated with the risk of RPP. Multivariate linear regression analysis showed that the serum hemoglobin A1c level (per 1% increase) measured at follow-up CCTA was independently associated with the annual change in the PAV (β: 0.098, 95% confidence interval [CI]: 0.048–0.149; P < 0.001). The multiple logistic regression models showed that the serum hemoglobin A1c level had an independent and positive association with the risk of RPP. The optimal predictive cut-off value of the hemoglobin A1c level for RPP was 7.05% (sensitivity: 80.0%, specificity: 86.7%; area under curve: 0.816 [95% CI: 0.574–0.999]; P = 0.017).

**Conclusion:**

In this retrospective case–control study, the glycemic control status was strongly associated with the risk of RPP in patients without a baseline coronary plaque burden. This suggests that regular monitoring of the glycemic control status might be helpful for preventing the rapid progression of coronary atherosclerosis irrespective of the baseline risk factors. Further randomized investigations are necessary to confirm the results of our study.

**Trial registration:**

ClinicalTrials.gov NCT02803411.

**Supplementary Information:**

The online version contains supplementary material available at 10.1186/s12933-022-01656-9.

## Background

Rapid progression of coronary atherosclerosis is strongly associated with a higher risk of future cardiovascular (CV) events [1, 2]. However, drawing clear associations between plaque progression and CV events is difficult due to complex interplays between various attributable factors, such as clinical comorbidities, medication usage, and characteristics of coronary plaques at the baseline [3–5]. The recent study of the Progression of Atherosclerotic Plaque Determined by Computed Tomographic Angiography Imaging (PARADIGM) registry revealed that among clinical, laboratory, and qualitative plaque features, the baseline coronary plaque burden is the most important risk factor for a rapid plaque progression (RPP) in the coronary arteries [6]. This indicates the significance of early detection of subclinical coronary atherosclerosis in an era that is focused on primary prevention. However, there is a paucity of data on associations of clinical variables with the risk of RPP in major epicardial coronary arteries in the absence of a baseline coronary plaque burden. Studies have provided firm evidence in favor of the usefulness of coronary computed tomography angiography (CCTA) for the non-invasive assessment of coronary artery disease (CAD) due to its ability to evaluate changes in coronary atherosclerosis through serial examinations [7, 8]. Accordingly, this study aimed to investigate the predictors for RPP in patients without baseline coronary plaques who underwent serial CCTA examinations.

## Methods

### Study design and patients

The PARADIGM registry has been described previously [9]. It is an international, prospective, and observational registry for evaluating associations between clinical factors and changes in coronary atherosclerosis using serial CCTA examinations. Between 2003 and 2015, 2,252 consecutive participants underwent CCTA at 13 centers across seven countries; images of interpretable quality, obtained by a 0.5 mm cross-sectional analysis in accordance with the Society of Cardiovascular Computed Tomography (SCCT) guidelines, were available for 1,760 of these patients [10, 11]. Baseline CCTA revealed no coronary plaques in 402 of these 1,760 patients; these were finally included in our analysis for identifying the predictors of RPP. Laboratory tests were performed within 1 month of all CCTA examinations; all blood samples were collected after at least 8 h of fasting. All methods in this study were performed in accordance with the relevant guidelines and regulations. This study was approved by the corresponding institutional review boards for each site.

### Acquisition and interpretation of CCTA images

CCTA examinations were performed twice (at the baseline and follow-up) using a scanner with ≥ 64-detector rows. Image acquisition and post-image processing were conducted in accordance with the SCCT guidelines [10, 11]. Datasets at both examinations were transferred to an offline workstation for image analysis with a semiautomated plaque analysis software (QAngioCT Research Edition v2.1.9.1; Medis Medical Imaging Systems, Leiden, the Netherlands) with manual correction [12]. All CCTA images were analyzed by independent level-III experienced readers who were blinded to the patients’ clinical data.

Segments with a diameter ≥ 2 mm were evaluated using a modified 17-segment model of the coronary arteries [10, 11]. Plaque volumes (mm^3^) were obtained for every coronary segment and summated to generate the total plaque volume on a per-patient basis. The total plaque volume was subclassified by composition using predefined and validated intensity cut-off values in Hounsfield units (HU); these classifications were as follows: necrotic-core plaques (− 30 to 30 HU), fibro-fatty plaques (31–130 HU), fibrous plaques (131–350 HU), and calcified plaques (≥ 351 HU) [13, 14]. The corresponding coronary segments were registered together using fiduciary landmarks (including branch vessel takeoffs and distance from the ostia) to compare atherosclerotic changes on CCTA images between the baseline and follow-up. The percentage atheroma volume (PAV) (%) was defined as the total plaque volume divided by the total vessel volume [15]. RPP was defined as an annual change of ≥ 1.0%/year in the PAV [6, 16].

### Statistical analysis

Continuous variables are expressed as mean ± standard deviation, while categorical variables are expressed as absolute values and proportions. Continuous variables were compared between baseline and follow-up CCTA using a paired t-test. Linear regression analysis was used to assess the associations between clinical variables and the annualized total PVC. Logistic regression analysis was used to identify the associations of clinical variables with the risk of RPP. Variables with P < 0.05 in the univariate analysis were considered as confounders and were included into the multivariate regression analysis. Considering the incidence of RPP, the number of independent variables that were included in the multivariate logistic regression analysis was strictly limited. With the exception of the baseline traditional risk factors and non-modifiable factors (age and sex), other independent variables measured at follow-up CCTA were included in the regression analyses. A receiver operating characteristic (ROC) curve analysis was performed using the Youden index to determine the optimal cut-off values of independent variables for RPP prediction. All statistical analyses were performed using the Statistical Package for the Social Sciences version 19 (IBM Corp, Armonk, New York, USA). P < 0.05 was considered significant for all analyses.

## Results

### Clinical characteristics

The clinical characteristics of the patients at baseline and follow-up CCTA are presented in Table 1. The mean age was 57.6 ± 10.0 years, and 198 patients (49.3%) were men. At baseline CCTA, the prevalence of hypertension, diabetes mellitus, hyperlipidemia, obesity, and current smoking status in the study population was 43.0%, 12.9%, 34.1%, 43.0%, and 14.4%, respectively. Newly developed diabetes mellitus and coronary plaques at follow-up CCTA were identified in 24 (6.0%) and 143 (35.6%) patients, respectively. Furthermore, statin use was observed in 30.0% and 45.3% of the patients at baseline and follow-up CCTA, respectively. The overall incidence of RPP was 4.2%. The annual plaque volume changes for each coronary plaque subtype according to statin use are described in **Additional File 1** (Table S1).

**Table 1:** Clinical characteristics

Characteristics	Total (n = 402)
Age, years	57.6 ± 10.0
Traditional risk factors, n (%)	
Advanced age (≥ 60 years)	172 (42.8)
Male	198 (49.3)
Hypertension	173 (43.0)
Diabetes mellitus	52 (12.9)
Metformin use	20 (5.0)
Insulin use	6 (1.5)
Hyperlipidemia	137 (34.1)
Obesity	173 (43.0)
Current smoking	58 (14.4)
At baseline CCTA	
BMI, kg/m^2^	24.9 ± 3.2
SBP, mmHg	127.1 ± 18.5
DBP, mmHg	77.2 ± 10.9
Total cholesterol, mg/dL	189.6 ± 41.3
Triglyceride, mg/dL	136.2 ± 75.4
HDL-C, mg/dL	51.7 ± 13.7
LDL-C, mg/dL	116.3 ± 33.8
Creatinine, mg/dL	0.98 ± 0.73
Glucose, mg/dL	105.4 ± 31.9
Hemoglobin A1C, %	6.3 ± 1.3
Statin use, n (%)	121 (30.0)
At follow-up CCTA	
BMI, kg/m^2^	25.1 ± 3.3
SBP, mmHg	125.8 ± 17.6
DBP, mmHg	76.3 ± 10.5
Total cholesterol, mg/dL	178.8 ± 38.7*
Triglyceride, mg/dL	135.1 ± 93.7
HDL-C, mg/dL	50.6 ± 13.5*
LDL-C, mg/dL	105.4 ± 31.9*
Creatinine, mg/dL	0.90 ± 0.50*
Glucose, mg/dL	106.7 ± 33.7
Hemoglobin A1C, %	6.3 ± 1.1
Statin use, n (%)	182 (45.3)
Follow-up CCTA findings	
Newly developed plaque, n (%)	143 (35.6)
RPP, n (%)	17 (4.2)
Plaque volume, mm^3^	
Total	12.98 ± 32.71
Fibrous	7.04 ± 19.62
Fibrous-fatty	2.99 ± 10.23
Necrotic core	0.44 ± 2.63
Dense calcium	2.49 ± 8.90

Values are given as mean ± standard deviation or number (%)

*P < 0.05 vs. baseline CCTA in laboratory findings

*BMI* body mass index, *CCTA* coronary computed tomography angiography, *DBP* diastolic blood pressure, *HDL-C* high-density lipoprotein cholesterol, *LDL-C* low-density lipoprotein cholesterol, *RPP* rapid plaque progression, *SBP* systolic blood pressure

### Clinical factors and coronary atherosclerotic changes

The associations between the baseline traditional risk factors and the annual change in total PAV and RPP are presented in Table 2. Advanced age (≥ 60 years), hypertension, diabetes mellitus, hyperlipidemia, obesity, and current smoking status were not significantly associated with either the annual change in total PAV or RPP. Table 3 shows the association of clinical variables with the annual change in total PAV. Univariate regression analysis revealed that the serum high-density lipoprotein cholesterol (HDL-C), low-density lipoprotein cholesterol (LDL-C), and hemoglobin A1c levels as well as statin use were significantly associated with the annual change in the PAV. Multivariate regression analysis revealed that the serum hemoglobin A1c level (per 1% increase) was independently and positively associated with the annual change in total PAV (β: 0.098, 95% confidence interval [CI]: 0.048–0.149; P < 0.001). The associations between the serum hemoglobin A1c level and the annual plaque volume changes in each coronary plaque subtype are described in Additional file 2 (Table S2).

**Table 2:** Associations of baseline traditional risk factors with annual changes in total PAV and RPP

	Annual change of total PAV			RPP		
	Β	95% CI	P	OR	95% CI	P
Advanced age (≥ 60 years)	0.053	‒0.029 to 0.135	0.208	2.551	0.924–7.039	0.071
Male	0.058	‒0.023 to 0.139	0.159	1.497	0.558–4.014	0.423
Hypertension	0.069	‒0.013 to 0.151	0.097	1.928	0.719–5.173	0.192
Diabetes mellitus	0.075	‒0.045 to 0.196	0.220	1.461	0.405–5.266	0.563
Hyperlipidemia	‒0.011	‒0.096 to 0.075	0.805	0.583	0.186–1.823	0.354
Obesity	‒0.067	‒0.150 to 0.017	0.116	0.379	0.121–1.183	0.095
Current smoking	0.019	‒0.097 to 0.134	0.752	1.274	0.354–4.579	0.711

*CI* confidence interval, *OR* odds ratio, *PAV* percentage atheroma volume, *RPP* rapid plaque progression

**Table 3:** Associations of clinical variables with the annual change in total PAV

	Univariate			Multivariate		
	β	95% CI	P	β	95% CI	p
Age at enrollment, per 1 year increase	0.004	‒0.001 to 0.008	0.073			
BMI, per 1 kg/m^2^ increase	‒0.006	‒0.019 to 0.006	0.317			
SBP, per 10 mmHg increase	0.016	‒0.008 to 0.040	0.202			
DBP, per 10 mmHg increase	0.018	‒0.023 to 0.058	0.394			
Triglyceride, per 10 mg/dL increase	‒0.003	‒0.008 to 0.002	0.209			
HDL-C, per 10 mg/dL increase	‒0.046	‒0.080 to -0.013	0.007	‒0.028	‒0.070 to 0.015	0.204
LDL-C, per 10 mg/dL increase	‒0.021	‒0.035 to -0.007	0.004	‒0.004	‒0.022 to 0.013	0.650
Creatinine, per 1 mg/dL increase	‒0.041	‒0.137 to 0.055	0.401			
Hemoglobin A1C, per 1% increase	0.117	0.070–0.164	< 0.001	0.098	0.048–0.149	< 0.001
Statin use	0.118	0.034–0.201	0.006	0.078	‒0.036 to 0.192	0.176

With the exception of age, independent variables were measured at follow-up CCTA.

*BMI* body mass index, *CCTA* coronary computed tomography angiography, *CI* confidence interval, *DBP* diastolic blood pressure, *HDL-C* high-density lipoprotein cholesterol, *LDL-C* low-density lipoprotein cholesterol, *PAV* percentage atheroma volume, *SBP* systolic blood pressure

### Association of serum hemoglobin A1c level with the risk of RPP

**Fig. 1:**
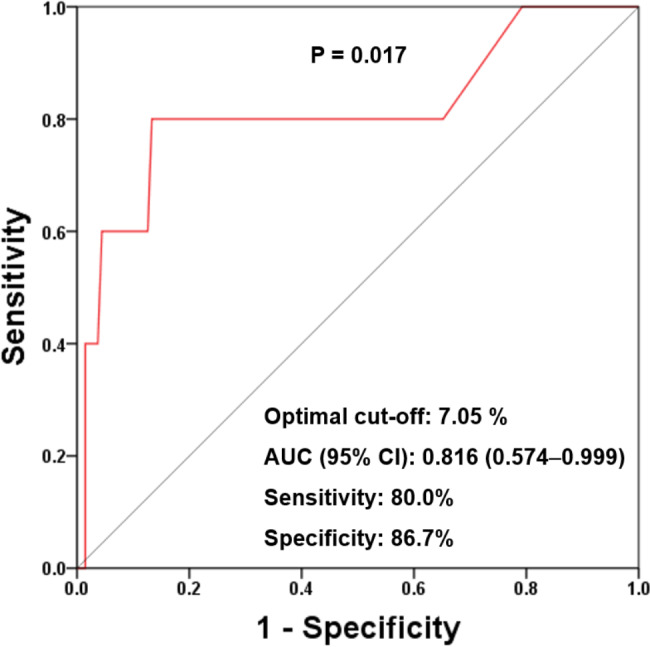
Receiver operating characteristic curve with respect to the serum hemoglobin A1c level for predicting RPP. RPP: rapid plaque progression *RPP*: rapid plaque progression

The univariate regression analysis showed that the serum hemoglobin A1c level (per 1% increase) was significantly associated with the risk of RPP (odds ratio: 2.384, 95% CI: 1.383–4.110; P = 0.002) (Additional file 3: Table S3). The multiple regression models showed that the serum hemoglobin A1c level had an independent and positive association with the risk of RPP (Table 4). ROC curve analysis revealed that the optimal cut-off value of the serum hemoglobin A1c level for predicting RPP was 7.05%, with a sensitivity and specificity of 80.0% and 86.7%, respectively (area under curve: 0.816, 95% CI: 0.574–0.999; P = 0.017; Fig. 1).

**Table 4:** Association between serum hemoglobin A1c level (per 1% increase) and the risk of RPP

	OR	95% CI	P
Model 1	2.500	1.322–4.725	0.005
Model 2	2.619	1.325–5.178	0.006
Model 3	2.216	1.122–4.378	0.022
Model 4	2.654	1.416–4.975	0.002
Model 5	2.069	1.160–3.691	0.014
Model 6	2.393	1.208–4.741	0.012

With the exception of age, independent variables were measured at follow-up CCTA.

Model 1 = adjusted for age and HDL-C level; Model 2 = adjusted for age and LDL-C level; Model 3 = adjusted for age and statin use; Model 4 = adjusted for HDL-C and LDL-C levels; Model 5 = adjusted for HDL-C level and statin use; Model 6 = adjusted for LDL-C level and statin use

*BMI* body mass index, *CCTA* coronary computed tomography angiography, *CI* confidence interval, *DBP* diastolic blood pressure, *HDL-C* high-density lipoprotein cholesterol, *LDL-C* low-density lipoprotein cholesterol, *OR* odds ratio, *RPP* rapid plaque progression, *SBP* systolic blood pressure

## Discussion

Data on the history of coronary atherosclerosis in subjects without any coronary plaques at baseline has been limited. This is related to the fact that the performance of CCTA in this population is not yet justified despite the significant advances of CCTA technique. The present study was possible to evaluate this issue because the PARADIGM is, to the best of our knowledge, the largest serial CCTA registry to date. This is the first study with longitudinal quantitative assessment of the major epicardial coronary arteries to identify the significance of glycemic control status on the risk of RPP at follow-up CCTA in patients without evidence of coronary plaques on baseline CCTA images. In our study, the optimal predictive cut-off value of hemoglobin A1c level for RPP was same as that for defining uncontrolled diabetes in clinical practice [17]. These findings demonstrate the importance of regular monitoring of the glycemic control status (and not just of the baseline traditional risk factors) for preventing the rapid progression of coronary atherosclerosis.

Using a machine learning framework, a recent study identified quantitative atherosclerosis characterization as the most important factor for identifying patients at a risk of RPP (beyond traditional clinical, laboratory, and qualitative atherosclerotic findings) [6]. Numerous previous studies have identified the clinical implications of a baseline coronary artery calcium score (CACS) of zero and the progression of coronary artery calcification in an asymptomatic general population [18–20]. Although a baseline CACS of zero has a long-term warranty period against mortality in patients at a low-to-intermediate CV risk, it does not reflect the presence of non-calcified coronary plaques. Moreover, data on changes in coronary atherosclerosis in patients with no coronary plaques at the baseline, especially with respect to RPP, are limited. This may be related to the fact that serial CCTA examinations have not yet been justified for this population, despite remarkable advances in CCTA techniques. For the current study, it was possible to evaluate changes in coronary atherosclerosis in patients with no coronary plaques at the baseline because of the PARADIGM registry, which is the largest serial CCTA registry to date.

Diabetes is strongly associated with an increased risk of severe CAD and subsequent CV events, even in asymptomatic patients [21, 22]. Kim et al. previously reported that patients with diabetes experience a greater plaque progression with adverse plaque formation than those without diabetes [23]. In addition, PARADIGM sub-studies found that insulin resistance estimated by the triglyceride glucose index and the atherogenic index of plasma was associated with an increased risk of RPP [24, 25]. However, recent data have suggested that these parameters may not be independently predictive of coronary atherosclerosis progression in patients with an advanced CAD at the baseline [26, 27]. Considering that the baseline coronary plaque volume increased with increases in the levels of these parameters in the PARADIGM sub-studies, it is possible that the baseline plaque burden influences the risk of RPP. In our study, 35.6% of the patients without coronary plaques at baseline CCTA presented with newly developed coronary plaques at follow-up CCTA (performed after a median of 3.6 years from the baseline); this suggested that subclinical coronary atherosclerosis progressed almost inevitably with a limited effect on the CV risk factors [28]. Although the baseline traditional risk factors, including advanced age, hypertension, diabetes mellitus, hyperlipidemia, obesity, and current smoking status, were not predictive of RPP development, the serum hemoglobin A1c level at follow-up was positively associated with the annual change in the PAV and the risk of RPP after adjusting for confounders. These results imply that new-onset diabetes and the glycemic control status in patients with diabetes have a substantial effect on the development of RPP in the absence of a baseline coronary plaque burden. Recent observational data from the Progression of Early Subclinical Atherosclerosis study revealed that higher serum hemoglobin A1c levels were associated with an increased risk of subclinical atherosclerosis even at the pre-diabetic stage [29]. Accordingly, further prospective investigations with larger sample sizes are necessary to confirm both the relationship between glycemic control and RPP in patients with diabetes and the significance of glycemic control according to the diabetes status.

Changes in the coronary atherosclerotic plaque composition and subtypes are affected by diverse clinical factors [8, 23, 30]. However, there is a paucity of data on newly developed coronary plaque subtypes during plaque progression among patients without baseline coronary plaques. The development of fibrous plaques was predominant in the overall study population in the present study. We found that higher serum hemoglobin A1c levels at follow-up were positively associated with annual changes in the volumes of fibrous and dense calcium plaques. In addition, annual changes in the calcified plaque volume differed significantly according to statin use. Further investigations are necessary to determine the significance of our findings in clinical practice.

The present study has some limitations. First, we only included patients without coronary plaques on baseline CCTA images from the PARADIGM registry. Therefore, the characteristics of our study population did not represent the overall characteristics of the patients in the PARADIGM registry. Second, data on consecutive changes in the clinical variables during follow-up periods were unavailable. Third, medications were not controlled because of the observational nature of the study. Furthermore, data on the glucose control methods at follow-up were also unavailable. Fourth, we were unable to confirm the effect of the glycemic control status on small coronary arteries. Finally, atherosclerotic findings could have been affected by the HU density despite the application of strict and standardized criteria for CCTA examination. However, this was the first study to use serial CCTA examinations to identify the impact of an optimal glycemic control on the risk of RPP in major epicardial coronary arteries in patients with no coronary plaques at the baseline.

## Conclusion

This retrospective case–control study showed the independent association between glycemic control and the risk of RPP using serial quantitative CCTA assessments during the near-term period amongst patients without coronary plaques on baseline CCTA images. Further prospective and randomized studies with larger sample sizes and longer follow-up durations should be conducted to confirm these results. Regular monitoring of the glycemic control status might be helpful in preventing the rapid progression of coronary atherosclerosis, irrespective of the baseline risk factors.

## Electronic supplementary material

Below is the link to the electronic supplementary material.

**Additional file 1**: Table S1 Comparison of the annual plaque volume changes for each coronary plaque subtype according to statin use
**Additional file 2**: Table S2 Association of the serum hemoglobin A1C level (per 1% increase) with the annual plaque volume changes for each coronary plaque subtype
**Additional file 3**: Table S3 Univariate logistic regression analysis for the associations of clinical variables with the risk of RPP

## Data Availability

The datasets used and analyzed during the current study are available from the corresponding author on reasonable request.

## Abbreviations

BMI: body mass index
CACS: coronary artery calcium score
CAD: coronary artery disease
CCTA: coronary computed tomography angiography
CI: confidence interval
CV: cardiovascular
DBP: diastolic blood pressure
HDL-C: high-density lipoprotein cholesterol
LCL-C: low-density lipoprotein cholesterol OR, odds ratio
PAV: percentage atheroma volume
PARADIGM: progression of atherosclerotic plaque determined by computed tomographic angiography imaging
RPP: rapid plaque progression
SBP: systolic blood pressure
