# Supplementary material for: Glycemic control is independently associated with rapid progression of coronary atherosclerosis in the absence of a baseline coronary plaque burden: a retrospective case–control study from the PARADIGM registry
Source: Cardiovasc Diabetol. 2022 Nov 12;21:239. doi: 10.1186/s12933-022-01656-9 (PMC9655903; doi:10.1186/s12933-022-01656-9)
Supplement: Supplementary file 1 — Additional file 1: Table S1 Comparison of the annual plaque volume changes for each coronary plaque subtype according to statin use Additional file 2: Table S2 Association of the serum hemoglobin A1C level (per 1% increase) with the annual plaque volume changes for each coronary plaque subtype Additional file 3: Table S3 Univariate logistic regression analysis for the associations of clinical variables with the risk of RPP [file 12933_2022_1656_MOESM1_ESM.docx]

**Supplementary materials**

**Table S1** Comparison of the annual plaque volume changes for each coronary plaque subtype according to statin use.

|  | No statin use | Statin use | P |
| --- | --- | --- | --- |
| Fibrous, mm^3^/year | 1.42 ± 3.96 | 2.11 ± 4.67 | 0.121 |
| Fibrous-fatty, mm^3^/year | 0.66 ± 2.16 | 0.89 ± 3.00 | 0.393 |
| Necrotic-core, mm^3^/year | 0.08 ± 0.45 | 0.14 ± 0.82 | 0.353 |
| Dense calcium, mm^3^/year | 0.31 ± 1.06 | 0.79 ± 1.98 | 0.004 |

Values are given as mean ± standard deviation.

**Table S2** Association of the serum hemoglobin A1c level (per-1 % increase) with the annual plaque volume changes for each coronary plaque subtype.

|  | Univariate | | |
| --- | --- | --- | --- |
|  | β | 95% CI | p |
| Fibrous, mm^3^/year | 0.723 | 0.245–1.201 | 0.003 |
| Fibrous-fatty, mm^3^/year | -0.058 | -0.337 to 0.221 | 0.683 |
| Necrotic core, mm^3^/year | -0.019 | -0.063 to 0.026 | 0.410 |
| Dense calcium, mm^3^/year | 0.698 | 0.464–0.932 | <0.001 |

*CI* confidence interval.

**Table S3** Univariate logistic regression analysis for the associations of clinical variables with the risk of RPP

|  | OR | 95% CI | P |
| --- | --- | --- | --- |
| Age at enrollment, per 1 year increase | 1.066 | 1.011–1.123 | 0.018 |
| BMI, per 1 kg/m^2^ increase | 0.933 | 0.783–1.112 | 0.439 |
| SBP, per 10 mmHg increase | 1.246 | 0.949–1.635 | 0.114 |
| DBP, per 10 mmHg increase | 1.199 | 0.725–1.984 | 0.480 |
| Triglyceride, per 10 mg/dL increase | 0.937 | 0.851–1.033 | 0.191 |
| HDL-C, per 10 mg/dL increase | 0.621 | 0.396–0.972 | 0.037 |
| LDL-C, per 10 mg/dL increase | 0.831 | 0.696–0.992 | 0.040 |
| Hemoglobin A1c, per 1 % increase | 2.384 | 1.383–4.110 | 0.002 |
| Creatinine, per 1 mg/dL increase | 0.074 | 0.005–1.204 | 0.067 |
| Statin use | 4.974 | 1.394–17.752 | 0.013 |

With the exception of age, other independent variables were measured at follow-up CCTA.

*BMI* body mass index, *CCTA* coronary computed tomography angiography, *CI* confidence interval, *DBP* diastolic blood pressure, *HDL-C* high-density lipoprotein cholesterol, *LDL-C* low-density lipoprotein cholesterol, *OR* odds ratio, *RPP* rapid plaque progression, *SBP* systolic blood pressure.
